# Murine DX5^+^NKT Cells Display Their Cytotoxic and Proapoptotic Potentials against Colitis-Inducing CD4^+^CD62L^high^ T Cells through Fas Ligand

**DOI:** 10.1155/2018/8175810

**Published:** 2018-09-30

**Authors:** Jens M. Werner, Michael Damian, Stefan A. Farkas, Hans J. Schlitt, Edward K. Geissler, Matthias Hornung

**Affiliations:** Department of Surgery, University Hospital Regensburg, University of Regensburg, Regensburg, Germany

## Abstract

**Introduction:**

It has been previously shown that immunoregulatory DX5^+^NKT cells are able to prevent colitis induced by CD4^+^CD62L^high^ T lymphocytes in a SCID mouse model. The aim of this study was to further investigate the underlying mechanism in vitro.

**Methods:**

CD4^+^CD62L^high^ and DX5^+^NKT cells from the spleen of Balb/c mice were isolated first by MACS, followed by FACS sorting and cocultured for up to 96 h. After polyclonal stimulation with anti-CD3, anti-CD28, and IL-2, proliferation of CD4^+^CD62L^high^ cells was assessed using a CFSE assay and activity of proapoptotic caspase-3 was determined by intracellular staining and flow cytometry. Extrinsic apoptotic pathway was blocked using an unconjugated antibody against FasL, and activation of caspase-3 was measured.

**Results:**

As previously shown in vivo, DX5^+^NKT cells inhibit proliferation of CD4^+^CD62L^high^ cells in vitro after 96 h coculture compared to a CD4^+^CD62L^high^ monoculture (proliferation index: 1.39 ± 0.07 vs. 1.76 ± 0.12; *P* = 0.0079). The antiproliferative effect of DX5^+^NKT cells was likely due to an induction of apoptosis in CD4^+^CD62L^high^ cells as evidenced by increased activation of the proapoptotic caspase-3 after 48 h (38 ± 3% vs. 28 ± 3%; *P* = 0.0451). Furthermore, DX5^+^NKT cells after polyclonal stimulation showed an upregulation of FasL on their cell surface (15 ± 2% vs. 2 ± 1%; *P* = 0.0286). Finally, FasL was blocked on DX5^+^NKT cells, and therefore, the extrinsic apoptotic pathway abrogated the activation of caspase-3 in CD4^+^CD62L^high^ cells.

**Conclusion:**

Collectively, these data confirmed that DX5^+^NKT cells inhibit proliferation of colitis-inducing CD4^+^CD62L^high^ cells by induction of apoptosis. Furthermore, DX5^+^NKT cells likely mediate their cytotoxic and proapoptotic potentials via FasL, confirming recent reports about iNKT cells. Further studies will be necessary to evaluate the therapeutical potential of these immunoregulatory cells in patients with colitis.

## 1. Introduction

It is well established that T cells, in particular naïve CD4^+^ T helper (Th) cells, play a key role in mediating immune responses and especially many aspects of autoimmune diseases [1–3]. In line with this hypothesis, liver damage in autoimmune hepatitis, for instance, is likely orchestrated by naïve CD4^+^ T cells recognizing an autoantigenic liver peptide [4]. In mice, it has been shown that transfer of enriched CD4^+^CD62L^high^ T cells into severe-combined-immunodeficient (SCID) mice induced chronic colitis [5–8]. For autoimmunity to occur, the antigen must be presented by antigen-presenting cells to naïve CD4^+^ T helper (Th0) cells. Once activated, Th0 cells can differentiate into Th1, Th2, or Th17 cells, initiating a cascade of immune reactions that are determined by the cytokines they produce [9].

In order to prevent effector cells to initiate and perpetuate tissue damage, subsequently resulting in autoimmune disease, there are several immune cell populations involved that tightly regulate their activation, such as regulatory T cells (Treg) [10] and NKT cells [11]. For instance, NKT cells prevent the development of experimental crescentic glomerulonephritis by inhibiting proliferation of mesangial cells [12] and they are able to inhibit the onset of type one diabetes by impairing the development of pathogenic T cells specifically targeting pancreatic beta cells [13]. There are also different cellular mechanisms involved, like the induction of programmed cell death to regulate respective immune responses in order to prevent self-endangering activities [14].

The acquisition of a distinct cytokine profile by naïve CD4^+^ T (Th0) cells and their proliferative capacity is modulated by specific cytokines. Th1 CD4^+^ T cell differentiation is mediated by IL-12 and IFN-*γ* that lead to the expression of the Th1 lineage specification transcription factor T-bet [15, 16]. Th2 cell differentiation depends on the activities of IL-4 and the transcription factor GATA3 [16]. Differentiation into each lineage is also opposed by cytokines; thus, IFN-*γ* promotes Th1 while suppressing Th2, IL-4 promotes Th2 and suppresses Th1, while TGF-*β* suppresses Th1 and Th2 cell differentiation [16].

Natural killer T (NKT) cells represent a subset of T lymphocytes that express NK cell markers such as NK1.1 and CD94, as well as T cell receptors (TCR) *α*/*β* with a restricted repertoire [17, 18]. These cells use a precisely rearranged homologous TCR variable (V) *α* and junctional (J) *α* segments. In mice, the invariant T cell receptor *α* chain V*α*14, encoded by J*α*18 with a conserved CDR3 region, is preferentially associated with V*β*8.2, V*β*7, or V*β*2 gene segments [17, 19]. In contrast to other T lymphocytes, the TCR of NKT cells only recognizes glycolipids presented by CD1d, which is a MHC class I-like glycoprotein that belongs to a group of CD1 molecules associated with *β*2-microglobulin [20–22]. Activation of NKT cells via CD1d antigen presentation initiates the production of both Th1 (IFN-*γ*) and Th2 cytokines (IL-4, IL-5, IL-13) [23] and increases their cytolytic activity [24].

Depending on CD1d reactivity and whether they either express or do not express the invariant V*α*14-J*α*18 TCR_*α*/*β*_, three subsets of NKT cells have been described. First, there are CD1d-dependent invariant NKT cells (iNKT) also called type I NKT cells. Second, a population of CD1d-reactive NKT cells expressing diverse TCR referred to as type II NKT cells has also been characterized. The third group consists NKT-like cells, which are CD1d-independent and express diverse TCR [18].

Several surrogate markers, such as NK1.1 in C57Bl/6 mice, coexpressed with the TCR_*α*/*β*_, have been used for identification of NKT cells [18]. Another frequently used marker for these cells in NK1.1^−^ mice strains is the antibody DX5, which recognizes the *α*_2_-integrin CD49b [25]. DX5 was initially characterized as a marker for NK cells [26], and more recently, DX5-coexpressing CD3^+^ lymphocytes have been described [27].

Previous studies, including from our group, revealed evidence for an immunoregulatory potency of DX5^+^NKT cells by the production of Th1 and Th2 cytokines [7, 28–31].

Although, all these data describe typical characteristics of NKT cells, there remains an ongoing discussion as to whether DX5^+^NKT cells belong to the class of CD1d-dependent NKT cells [32, 33]. However, less attention has been given to their cytotoxic potential so far.

In the present study, we further characterized an antiproliferative effect of DX5^+^NKT cells on colitis-associated CD4^+^CD62L^high^ cells. For this purpose, CD4^+^CD62L^high^ of the spleen of Balb/c mice was isolated and coculture experiments were set up with either DX5^+^NKT or CD8^+^ T cells. DX5^+^NKT cells had an antiproliferative effect and induced apoptosis in CD4^+^CD62L^high^ cells. Furthermore, we could identify Fas ligand (FasL) to be a key player in the cytotoxic and proapoptotic function of DX5^+^NKT cell potentials.

Consequently, we were able to show that the proapoptotic effect of DX5-NKT cells against CD4^+^CD62L^high^ T cells is directed by FasL. Our observation therefore confirms previous reports about the cytotoxicity of type I NKT cells and extends these to DX5-NKT cells [34, 35].

## 2. Methods

### 2.1. Cell Harvesting and Isolation

Different lymphocyte subsets were purified from splenic mononuclear cells isolated from Balb/c mice (Charles River Laboratories, Wilmington, MA, USA). If necessary, further isolation was performed by magnetic activated cell sorting (MACS; Miltenyi Biotec, Bergisch Gladbach, Germany) or by FACS (FACSAria I, BD Biosciences, San Jose, USA).

Briefly, cell suspension of the spleen was prepared by cutting small pieces and gently pressing through a 100 *μ*m wire mesh. DX5^+^ cells were purified using anti-mouse-DX5^+^ MicroBeads (Miltenyi Biotec). Cells were passed through a MACS column (type LS) attached to a MidiMACS magnet (Miltenyi Biotec). DX5^+^ cells were collected in the positive fraction. DX5^+^ splenocytes were labeled with FITC-conjugated anti-mouse CD3 molecular complex (clone: 17A2, rat IgG2b) and PE-conjugated anti-mouse CD49b (clone: DX5, rat IgM) (all from BD Biosciences) for further DX5^+^NKT cell isolation by FACS sorting. CD4^+^CD62L^high^ and CD4^+^CD62L_low_ cells were purified using the CD4^+^CD62L^+^ Isolation Kit (Miltenyi Biotec) and CD8^+^ cells by using anti-mouse-CD8^+^ MicroBeads (Miltenyi Biotec).

### 2.2. Antibodies and Flow Cytometry

The following reagents were used for cell labeling in multiparameter flow cytometric analysis (FACS Calibur, BD Biosciences): PE or FITC-conjugated anti-mouse CD4 (clone: RM4-5, Rat IgG2b) Alexa 648 or FITC-conjugated anti-mouse CD3 (clone: 17A2, rat IgG2b), Alexa 648 or FITC-conjugated anti-mouse-CD8a (clone: 53–6.7, rat IgG2a), PE-conjugated anti-mouse-CD49b (clone: DX5, rat IgM), FITC- or PE-conjugated anti-mouse-CD62L (clone: MEL-14, rat IgG2a), and FITC- or PE-conjugated anti-mouse-CD178 (Fas ligand) (clone: MFL3, hamster IgG1); all are from BD Biosciences. APC-conjugated anti-mouse-CD49b (clone: DX5, rat IgM) and FITC-conjugated anti-mouse-CD49b (clone: DX5, rat IgM) all are from Miltenyi Biotec. APC-conjugated anti-mouse-CD4 (clone: RM4-5, rat IgG2b) is from Caltag (Towcester, UK).

### 2.3. Coculture Experiments

Ninety-six well culture plates (Sigma-Aldrich, St. Louis, USA) were coated with anti-mouse-CD3e (clone: 145-2C11, BD Biosciences) at 10 *μ*g/ml and stored overnight at 4°C. As described in previous studies in more detail, after isolation, 2 × 10^5^ CD4^+^CD62L^high^ and CD4^+^CD62L_low_ cells were coincubated with either 2 × 10^5^ DX5^+^NKT cells or CD8^+^ T cells in 200 *μ*l RPMI culture medium (Gibco, Paisley, UK) in either coated or uncoated wells [7]. For further stimulation, 5 *μ*g/ml anti-mouse-CD28 (clone: 37.51, BD Biosciences) and 2000 IU/ml IL-2 (PeproTech, Rocky Hill, USA) were added [31]. Control cultures of CD4^+^CD62L^high^, CD4^+^CD62L_low_, DX5^+^NKT cells, and CD8^+^T cells only were incubated at 4 × 10^5^ cells in 200 *μ*l RPMI under the same conditions.

### 2.4. CFSE Proliferation Assay

After isolation, CD4^+^CD62L^high^ and low cells were labeled using the Vybrant CFDA SE Cell Tracer Kit (Molecular Probes, Eugene, USA). In brief, cells were incubated with 0.5 *μ*M CFSE solution for 15 min at 37°C. Pellet was washed once with culture medium to stop the staining reaction and then incubated for 60 min at room temperature to release excessive CFSE.

### 2.5. Intracellular Cytokine Staining

After cell isolation, cocultures were set up as mentioned above. Additionally, 50 ng/ml PMA (InvivoGen, San Diego, USA) was added from the beginning, 750 ng/ml ionomycin (Sigma-Aldrich, St. Louis, USA) for the last 4 h, and 1 *μ*g/ml GolgiPlug (BD Biosciences) was added 2 h before cell harvesting. Culture supernatants were harvested and stored at −20°C for IFN-*γ* ELISA. Cells were fixed in 1 ml Fix/Perm (eBioscience, Hatfield, UK) for 60 min at 4°C. After incubation with permeabilization buffer (eBioscience), cells were stained intracellular with PE-conjugated anti-mouse-Abs (IL-2, clone: JES6-5H4/IFN-*γ*, clone: XMG1.2/TNF-*α*, clone: MP6-XT22) from BD Biosciences and with PE-conjugated anti-mouse-IL-13 (clone: eBio13A) and FITC-conjugated anti-mouse-IFN-*γ* (clone: XMG1.2) all eBioscience.

### 2.6. Intracellular Caspase-3 Staining

After cell isolation, cocultures were set up as mentioned above. For 48 h coincubation, CD4^+^CD62L^high^ and CD4^+^CD62L_low_ cells were additionally labeled with CFSE. After the indicated time, cells were fixed in 1 ml Fix/Perm (eBioscience) for 60 min at 4°C. After incubation with permeabilization buffer (eBioscience), cells were stained intracellular with Alexa648-conjugated anti-mouse-caspase-3 (clone: C92–605, BD Biosciences). For FasL blocking (Kayagaki, Yamaguchi et al. 1997), DX5^+^NKT cells were preincubated with either 50 *μ*g/ml purified mouse-anti-FasL (clone: MFL4; BioLegend, Cambridge, UK) or 50 *μ*g/ml isotype control for 1 h and then cocultures were set up with CD4^+^CD62L^high^ cells as indicated above.

### 2.7. Statistics

All in vitro experiments were repeated at least 3 times, and data are presented as the mean value ± SEM. Statistical analyses were performed using either a Student's *t*-test or the Mann–Whitney *U* test. Differences were considered significant at *P* < 0.05.

## 3. Results

### 3.1. DX5^+^NKT Cells Have an Antiproliferative Effect on Colitis-Inducing CD4^+^CD62L^high^ Cells

**Figure 1:**
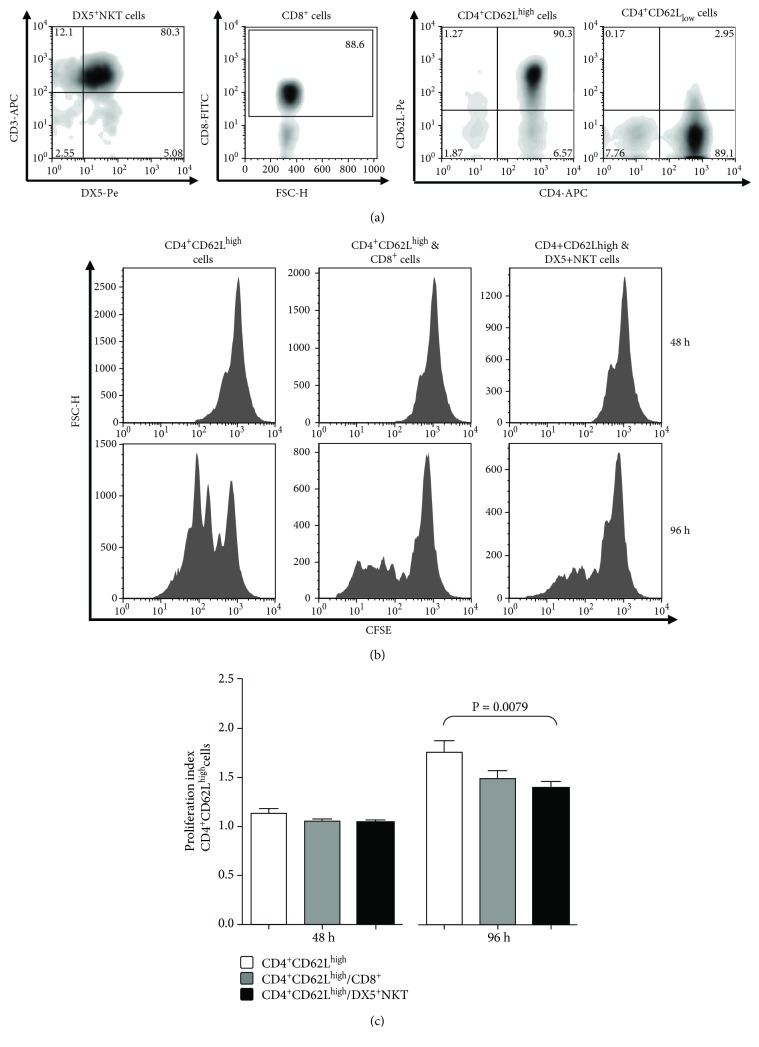
Flow cytometry analysis of the spleen CD3^+^DX5^+^NKT, CD8^+^ T, CD4^+^CD62L^high^, and CD4^+^CD62L_low_ cells of Balb/c mice after separation by MACS and FACS sorting (a). Proliferation (b) and proliferation index (c) of CFSE-labeled CD4^+^CD62L^high^ cells after 48 h and 96 h of monoculture or coculture with CD8^+^ T cells or CD3^+^ DX5^+^NKT. Results are given as mean + SEM. Experiments were repeated at least three times (^∗^*P* < 0.05).

Lymphocyte subsets, such as CD4^+^CD62L^high^ and CD8^+^T cells, were isolated from the spleen of Balb/c mice by MACS. DX5^+^NKT cells were isolated using MACS followed by FACS sorting (Figure 1(a)). First, we analyzed the proliferation of CD4^+^CD62L^high^ cells in coculture experiments with DX5^+^NKT and CD8^+^T cells (Figure 1(b)) using CFSE labeling. CD4^+^CD62L^high^ cells began to proliferate 48 h after stimulation with anti-CD3, anti-CD28, and IL-2. As shown in Figure 1(c), after 96 h coincubation with DX5^+^NKT cells, proliferation of CD4^+^CD62L^high^ cells significantly decreased compared to single cultures (proliferation index: 1.39± 0.07 vs. 1.76± 0.12; *P* = 0.0079). The antiproliferative effect of CD8^+^T cells was less distinctive and statistically not significant.

### 3.2. Decrease of IFN-*γ* Cytokine Secretion of CD4^+^CD62L^high^ Cells in Coculture Experiments with DX5^+^NKT Cells

**Figure 2:**
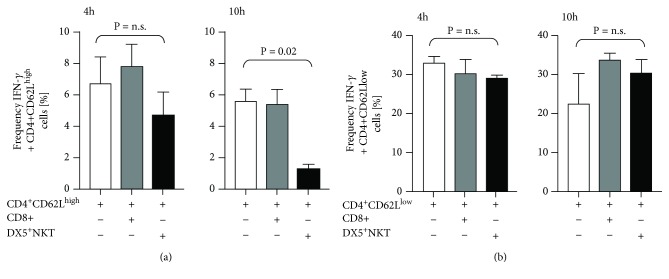
Intracellular flow cytometry detection of IFN-*γ* in CD4^+^CD62L^high^ (a) and CD4^+^CD62L_low_ cells (b) after 4 h and 10 h of monoculture or coculture with CD8^+^ T cells or CD3^+^DX5^+^NKT. Results are given as mean + SEM. Experiments were repeated at least three times (^∗^*P* < 0.05).

Next, we wanted to assess whether this antiproliferative effect was associated with differences in cytokine secretion. Therefore, the production of Th1 such as IFN-*γ*, TNF-*α*, and IL-2 and the Th2 cytokine IL-13 in CD4^+^CD62L^high^ cells was compared. Isolated CD4^+^CD62L^high^ cells were cultured for 4 and 10 h in the presence of anti-CD3 and anti-CD28 antibodies in a single or coculture with DX5^+^NKT or CD8^+^T cells, respectively. The cells were additionally incubated with PMA, ionomycin, and GolgiPlug for intracellular cytokine staining. As shown in Figure 2(a), after 10 h, CD4^+^CD62L^high^ cells produced the same amount of IFN-*γ* in a coculture with CD8^+^ cells as in a single culture (5.4% ± 0.95% vs. 5.6% ± 0.76%), whereas cocultured with DX5^+^NKT cells, IFN-*γ* production of CD4^+^CD62L^high^ cells was decreased (5.6% ± 0.76% vs. 1.3% ± 0.26%; *P* = 0.02). In contrast, looking at TNF-*α*, IL-2, and IL-13, no interference of DX5^+^NKT cells in cytokine production of CD4^+^CD62L^high^ cells could be detected. Furthermore, there was no significant effect looking at cocultures with CD4^+^CD62L_low_ cells (Figure 2(b)).

### 3.3. DX5^+^NKT Cells Activate Proapoptotic Caspase-3 in Colitis-Associated CD4^+^CD62L^high^ Cells

**Figure 3:**
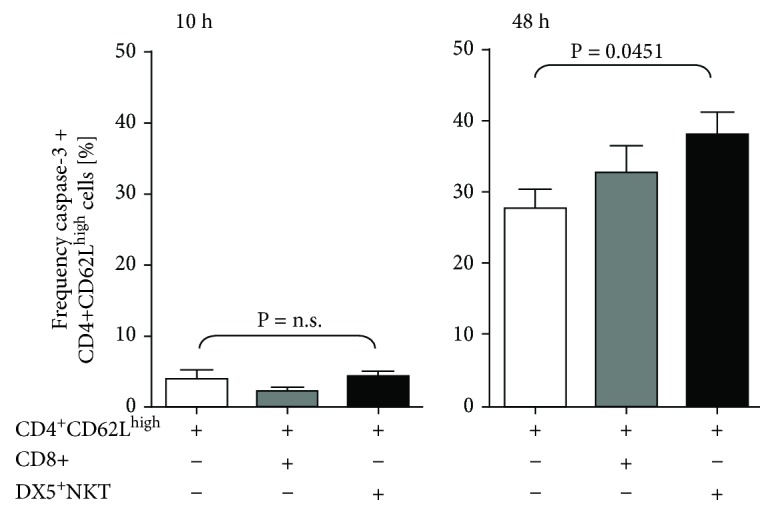
Intracellular flow cytometry analysis of caspase-3 in CD4^+^CD62L^high^ cells after 10 h and 48 h of monoculture or coculture with CD8^+^ T or CD3^+^DX5^+^NKT cells. Results are given as mean + SEM. Experiments were repeated at least three times (^∗^*P* < 0.05).

Results thus far display differences in the cytokine profile between CD8^+^ cells and DX5^+^NKT cells but cannot explain the antiproliferative effect on CD4^+^CD62L^high^ cells. Therefore, we wanted to assess whether the cytotoxic potential of DX5^+^NKT cells is involved in this process. We measured the amount of intracellular caspase-3 after 10 and 48 h coincubation of CD4^+^CD62L^high^ cells with either CD8^+^ cells or DX5^+^NKT cells. As shown in Figure 3, DX5^+^NKT cells significantly increased the number of caspase-3-positive cells after 48 h coculturing (38% vs. 28%; *P* = 0.0451) compared to the CD4^+^CD62L^high^ single culture.

### 3.4. FasL on DX5^+^NKT Cells Is Mediating the Induction of Proapoptotic Caspase-3 in Colitis-Associated CD4^+^CD62L^high^ Cells

**Figure 4:**
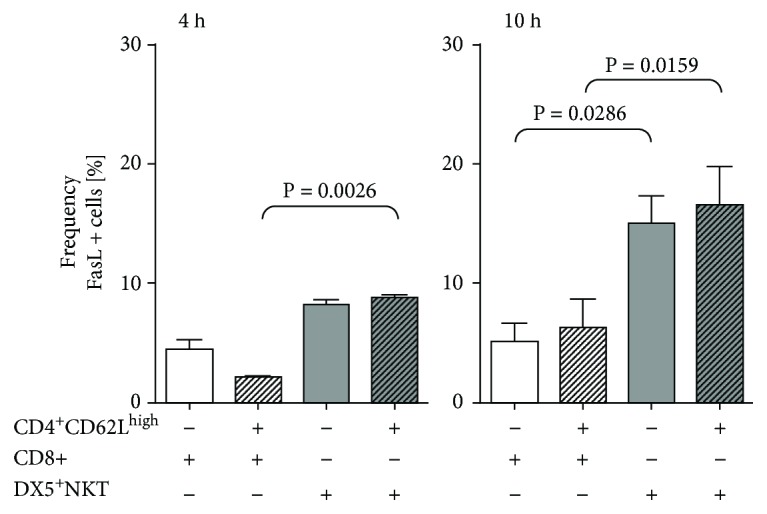
Flow cytometry analysis FasL expression of CD8 T and CD3^+^DX5^+^NKT cells after 4 h and 10 h monoculture or coculture with CD4^+^CD62L^high^ cells. Results are given as mean + SEM. Experiments were repeated at least three times (^∗^*P* < 0.05).

Next, we wanted to assess which route DX5^+^NKT cells used to induce caspase-3. Therefore, either CD8^+^ cells or DX5^+^NKT cells were cocultured with CD4^+^CD62L^high^ cells and stained for FasL expression. After 4 h, DX5^+^NKT cells in coculture expressed more FasL compared to CD8^+^ cells (9% vs. 2%; *P* = 0.0026). And after 10 h incubation, even more DX5^+^ NKT cells expressed FasL (16.6% ± 3.2%), whereas CD8^+^ cells in coculture with CD4^+^CD62L^high^ cells still expressed less FasL (6.3% ± 2.4%; *P* = 0.0159) compared to DX5^+^NKT cells (Figure 4).

**Figure 5:**
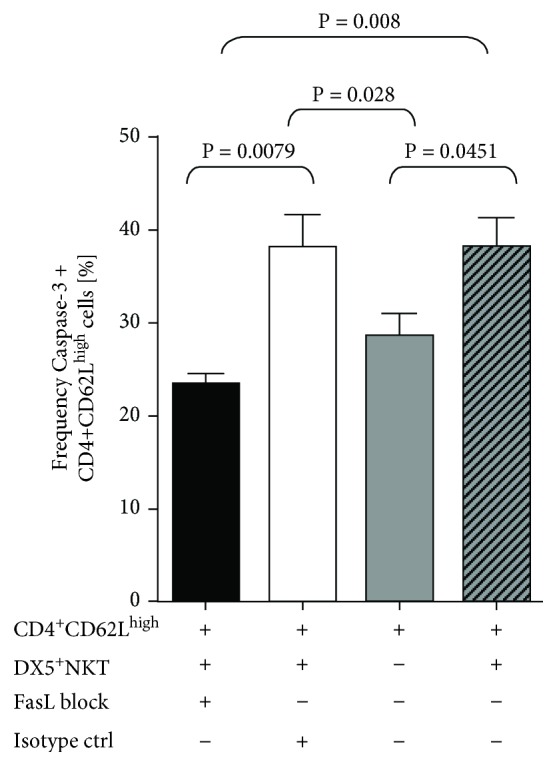
Intracellular flow cytometry analysis of caspase-3 in CD4^+^CD62L^high^ cells after 48 h of coculture with CD3^+^DX5^+^NKT cells and pretreatment with either FasL block or isotype control. Results are given as mean + SEM. Experiments were repeated at least three times (^∗^*P* < 0.05).

Finally, we wanted to prove that the induction of FasL expression is responsible for the activation of caspase-3 in CD4^+^CD62L^high^ cells. DX5^+^NKT cells were preincubated with a FasL blocking antibody for 1 h prior to coculturing with CD4^+^CD62L^high^ cells for 48 h. Additionally, cells were incubated with an isotype antibody for control reasons. As expected, coincubation with DX5^+^NKT cells increased the frequency of caspase-3-positive cells in the normal culture (34.4% ± 4.3%; *P* = 0.0451) as well as in the isotype control culture (38% ± 3.5%; *P* = 0.028) compared to a CD4^+^CD62L^high^ single culture (24% ± 1.5%). Interestingly, blocking of FasL on DX5^+^NKT cells reduced the frequency of caspase-3-positive CD4^+^CD62L^high^ cells in the coculture to that amount observed in the single culture (23.4% ± 1.2%; *P* = 0.008) (Figure 5).

## 4. Discussion

In this study, we show that proliferation of colitis-inducing CD4^+^CD62L^high^ T cells is prevented by DX5^+^NKT cells. After activation, naïve CD4^+^ cells can differentiate into Th1, Th2, or Th17 cells, initiating different immune reactions depending on their cytokine profile [2]. A variety of mechanisms are involved in regulation of CD4^+^ T cell function, including inhibition of proinflammatory cytokines, promoting increase of certain T cell populations and suppressing proliferation of different ones [9] [36]. Certain cytokines can suppress and induce differentiation, i.e., of Th17 cells [37] [38] [39], but for some activation also, direct cell interaction with B cells is required [40]. Toes and his group showed that DX5^+^CD4^+^ T cells were able to direct CD4^+^ T cells towards IL-10 production through IL-4 [41], and in addition, these cells impair the function of CD4^+^ T cells through specific inhibition of dendritic cells [42]. DX5^+^CD4^+^ T cells are comparable to DX5^+^NKT cells because over 80% of isolated DX5^+^NKT cells are CD4 positive [31]. Nevertheless, reported studies could not show an inhibition of proliferation of CD4^+^ T cells by DX5^+^CD4^+^ T cells [41]. This might be explained by the fact that the used cells were OVA-specific CD4^+^ T cells [41] and therefore different compared to naïve CD4^+^CD62L^high^ T cells. Previously published data of our group already revealed a decrease of colitis-inducing CD4^+^CD62L^high^ T cells after coculturing with DX5^+^NKT cells [7], and consistent in both settings, DX5^+^ T cells decreased IFN-*γ* production of OVA-specific CD4^+^ T cells as well as of CD4^+^CD62L^high^ T cells as shown in our study [41, 42]. However, we used CD8^+^ T cells to exclude an unspecific effect between the cultured subsets. This worked very well as a control for the IFN-gamma production assays but not so well, for example, in the proliferation assays. Furthermore, stimulation with IL-2 might also impact the responsiveness of CD4^+^CD62L^high^ cells, CD8^+^ T cells, and NKT cells at different levels. Furthermore, as shown before, DX5^+^NKT cells express in less 20% CD25 [31]. Therefore, a relatively small contribution of Tregs on the observed effects should be considered.

We found out that the inhibition of CD4^+^CD62L^high^ T cells proliferation after coculturing with DX5^+^NKT cells is associated with an increase of caspase-3 in CD4^+^CD62L^high^ T cells. Mature caspase-3 results from the processing of procaspase-3 and induces cell death through multiple cellular molecules. Caspase-3 is involved in both major apoptosis pathways: the intrinsic, which is mediated through the B cell lymphoma 2 (BCL-2) family, and the extrinsic pathway, which is induced through the interaction of FasL and Fas [14].

In our study, expression of FasL was significantly increased on DX5^+^NKT cells after 10 h especially in coculture with CD4^+^CD62L^high^ T cells. Furthermore, blockage of FasL resulted in a decrease of caspase-3 in CD4^+^CD62L^high^ T cells. These results are in agreement with a previous report from our group showing that DX5^+^NKT cells reduce colitis cells through PD-L1 in vitro. However, PD1, the counterpart of PD-L1, was expressed on both CD62L^high^ and CD62L_low_ cells, suggesting that PD-L1 killing activity is likely mediated via a different receptor [7]. FasL is known as a receptor on specific cytotoxic lymphocytes [43, 44], but until now, it has not been described on DX5^+^NKT cells. FasL's counterpart on the effector cell, Fas, is known to transmit signals that lead to cell death via apoptosis [45]. Our findings suggest that the decrease in the number of colitis-inducing CD4^+^CD62L^high^ T cells as well as their proliferation capacity in the presence of DX5^+^NKT cells is principally executed through FasL-Fas interaction. Loss of function of the FasL-Fas pathway can cause autoimmune diseases [46–49], and it is already known that FasL-Fas is required for elimination of T cells [50].

Taken these data together with our findings supposes that the interaction with DX5^+^NKT cells via FasL-Fas represents one mechanism in the regulation of the development of CD4^+^ T cells. Further studies should clear the importance of this effect in the origin of autoimmunity and the additional cofactors which are required to perform the function.

## Data Availability

All data supporting the results reported in the article are generated and archived in facilities of the Department of Surgery, University of Regensburg.
